# Fluorescence‐guided surgery for non‐melanoma and melanoma skin cancer: Case series and a brief review of the literature

**DOI:** 10.1111/phpp.12687

**Published:** 2021-05-07

**Authors:** Bobyr Ivan, Corradi Francesca, Rizzetto Giulio, Diotallevi Federico, Bianchelli Tommaso, Campanati Anna, Offidani Annamaria

**Affiliations:** ^1^ Dermatological Unit Department of Clinical and Molecular Sciences Polytechnic University of the Marche Region Ancona Italy

Surgery is the primary mode of treatment for non‐melanoma (NMSC) and melanoma skin cancer (MSC). The main purpose of surgery is to completely remove the neoplastic lesion, since the presence of residual tumor cells is considered a strong predictor of tumor recurrence, new surgical approach, and, therefore, survival.^1^

Fluorescence can be utilized to define tumor margins before and during surgery, improving the possibility of radical resection. More specifically, fluorescence‐guided surgery (FGS) has been applied to multiple surgical situations, as the identification of solid tumors and sentinel lymph node mapping.^1^

Protoporphyrin IX (PpIX) is the most widespread photosensitizer available in dermatology. In our case series, a topical cream containing a PpIX precursor (5‐aminolevulinic acid, ALA) is applied on the lesions plus a 2 cm margin for 3 hours. ALA is absorbed and enzymatically converted into PpIX by the heme biosynthesis pathway, naturally present in all nucleated cells. Neoplastic cells accumulate PpIX more rapidly than normal cells, because their heme biosynthesis is elevated, showing a more intense fluorescence than the surrounding normal skin.^2^ PpIX exhibits characteristic red fluorescence (at 635 nm and 700 nm) when excited by blue‐violet light (410 nm), and we used it to observe and define the edge of the lesions. The temperature of the room was 20°C, the fluorescence was evaluated in a dark room, and the photographs were taken with a reflex camera.

## 1 CASE SERIES

### 1.1 Case 1

**FIGURE 1:**
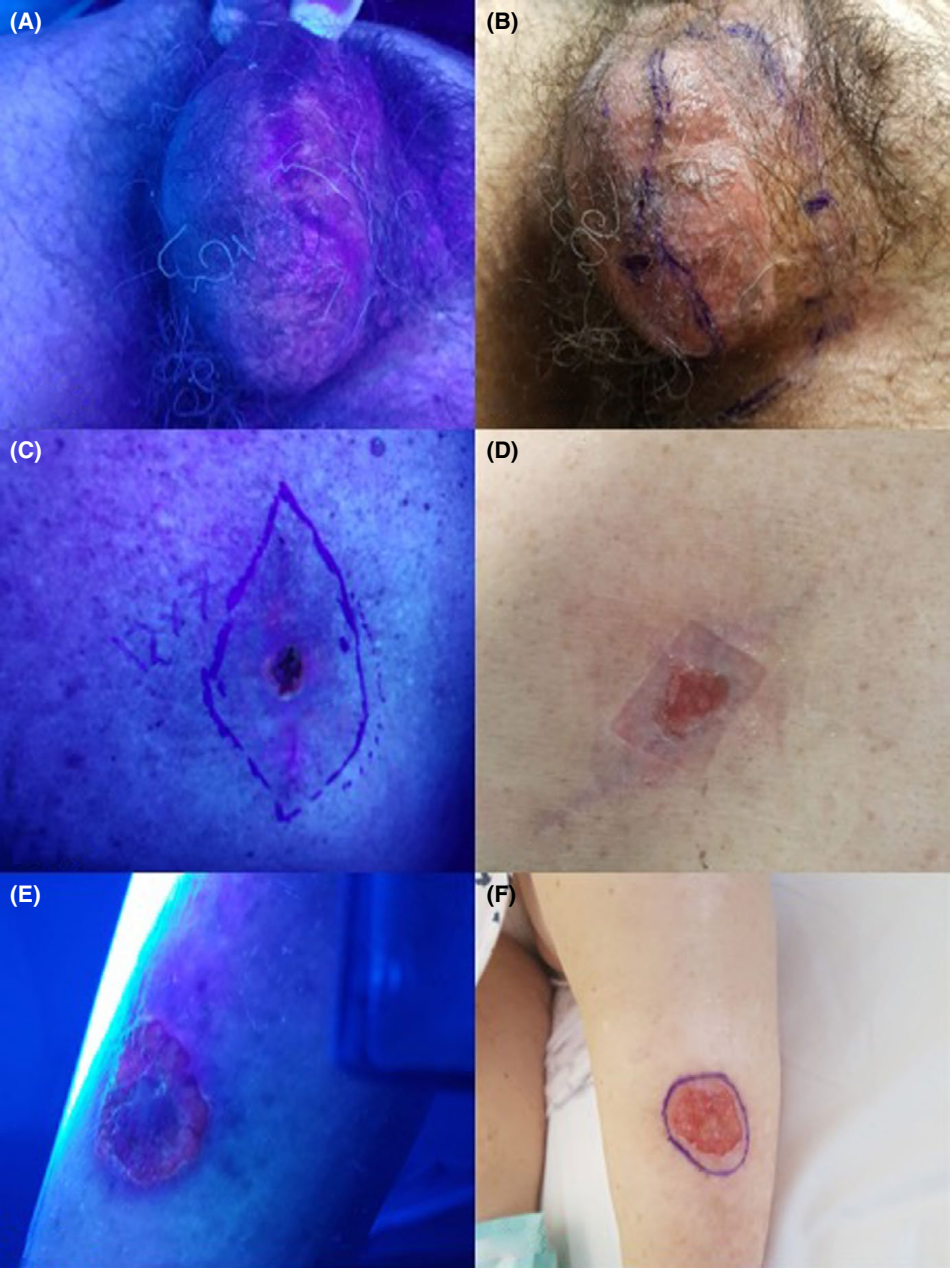
A, Scrotal extramammary Paget's disease (EMPD) at the fourth surgery. Wood's light and ALA fluorescence. B, New surgical margins. C, Basal‐cell carcinoma (BCC) of the back with vascular and nervous infiltration. He came to our clinic after two previous surgeries with positive edge. Wood's light and ALA fluorescence with the surgical margins. D, Clinical aspect. E, Squamous‐cell carcinoma (SCC) of the right leg and at the first surgery. Wood's light and ALA fluorescence. F, The new surgical margins

A 67‐year‐old (y.o) man with scrotal extramammary Paget's disease (EMPD) at the fourth surgery. The edge of the lesion in the first three surgeries was positive, while in the last surgery, after the FGS, was clear (Figure 1A,B).

### 1.2 Case 2

A 75‐year‐old man with basal‐cell carcinoma (BCC) of the back with vascular and nervous infiltration. He came to our clinic for the third surgery after two previous surgeries with positive edge. The last was completely clear (Figure 1C,D).

### 1.3 Case 3

A 80‐year‐old woman with squamous cell carcinoma (SCC) of the right leg, and at the first surgery, the edge of the lesion was clear (Figure 1E,F).

### 1.4 Case 4

**FIGURE 2:**
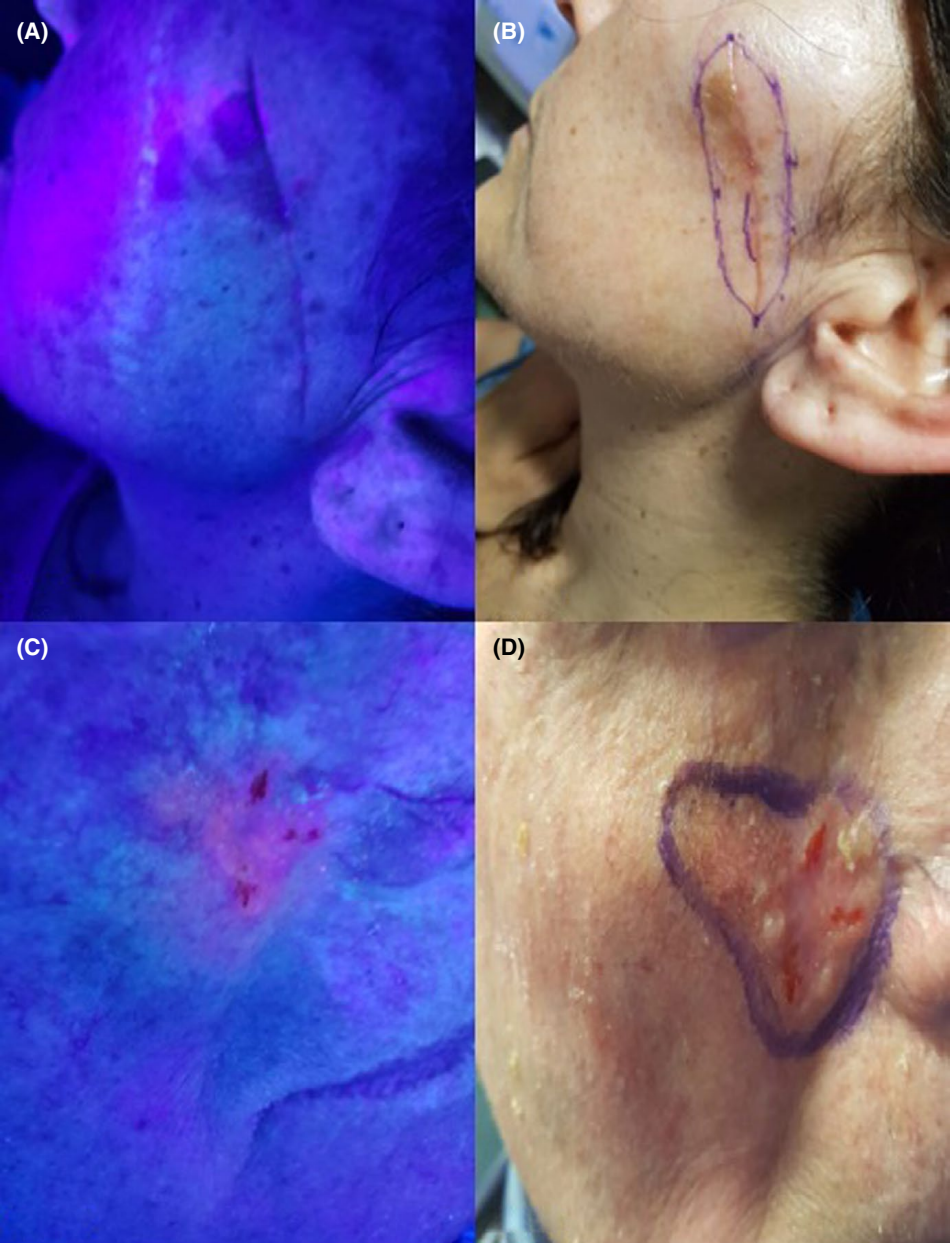
A, Lentigo Maligna of the face at the second surgery. Melanin photo‐interaction with Wood's light. B, The new surgical margins. C, Ulcerated BCC of the right medial cheek. Wood's light and ALA fluorescence. D, The new surgical margins

A 42‐year‐old woman with Lentigo Maligna of the face at the second surgery to clear the edge of the lesions. (Figure 2A,B) Dermoscopy showed asymmetric pigmentation in follicular opening and rhomboid structures.

### 1.5 Case 5

A 50‐year‐old man with ulcerated BCC of the right medial cheek and the edge was clear after the first surgery (Figure 2C,D).

## 2 DISCUSSION

The previously described method was already known as photodynamic diagnosis (PDD) and was successful in guiding the excision of BCC, SCC, and EMPD, while a clear utility in MSC had not been established.^3^

One of the main aims of skin surgery is the complete excision of NMSC and MSC. If the neoplasm is not totally included in the resection, a second enlargement procedure must be performed, with an increase in management costs and greater discomfort for the patient. Although many techniques have been proposed in the literature to achieve complete resection, as Mohs micrographic surgery and confocal microscopy,^4^ fluorescence can be more easily applied in the routine use since it is a widespread and accessible practice.

Fluorescence also allows to evaluate the Field of cancerization (FC), which is the area adjacent to visible skin lesions (SCC, BCC), constituted histologically by normal cells but with genetic alterations predisposing to the development of invasive neoplasms.^5^ The existence of the FC explains both local recurrences and the appearance of adjacent primitive tumors.^6^ Furthermore, if FC can be surgically resected, the risk of local recurrence and new lesions is considerably reduced. A limitation is the excessive extension of FC, which necessarily requires other treatments (PDT, topical ciclox inhibitors).^6^ In our case series, we identified BCC and SCC resection margins according to the distribution of fluorescence and histology always confirmed the complete excision.

We also evaluated the use of FGS on EMPD, which is a skin adenocarcinoma characterized by undefined margins, multicentricity, and high recurrence rate. ALA accumulates primarily in malignant cells, and its fluorescence is much stronger than that in normal skin.^7^ We used ALA for identifying the real extension of the disease and distant scattered lesions, obtaining a complete tumor‐free resection.

FGS can be also applied for Lentigo Maligna, which is an in situ tumor with atypical melanocytes, localized frequently on the head and neck, and characterized by indistinct margins. In our case, the lesion was particularly difficult to determine since the previous surgery scar. However, we managed to remove the lesion, exploiting the melanin photo‐interaction with Wood's light and ALA fluorescence. Only few studies^8^, ^9^ reported the use of fluorescence for determining surgical margins of melanocytic tumors, but its role can be very useful in all those lesions with unclear extension and superficial diffusion.

To our knowledge, this is the first study that reports the wide application of FGS in NMSC and MSC for both surgical intervention and re‐intervention. ALA was applied 2 cm beyond the macroscopic clinical boundaries to include the specific resection margins indicated by the guidelines.

Resection margins were adjusted according to fluorescence by exciding 2 mm over than the fluorescence margins. The histological extent of the tumor was found to be comparable with the fluorescent margin, as free margins of all the excised lesions were 2 mm or more.

In cases 3 and 5 (Figures 1E,F and 2C,D), the margin was reduced from that accepted for this type of lesion, and in cases 1, 2, and 4 (Figure 1A,B,C,D; 2A,B), it was enlarged, achieving complete excision in all cases (Table 1). The usefulness of FGS lies in the possibility of performing a complete resection of the lesion in a single surgical time, as it seems to make possible to adapt the resection margin in complex cases not easily identifiable on clinical base.

**TABLE 1:** Cases summarized

Case	Type of neoplasm	Change of surgical margin after FGS	Margin error*
1	Scrotal EMPD	Margin enlarged	+5 mm
2	BCC	Margin enlarged	+5 mm
3	SCC	Margin reduced	−3 mm
4	Lentigo Maligna	Margin enlarged	+6 mm
5	BCC	Margin reduced	−2 mm

Abbreviations: BCC, basal‐cell carcinoma; EMPD, extramammary Paget's disease; SCC, squamous‐cell carcinoma.

* Error between the clinical accepted margin for the type of tumor and the new margin after FGS.

In conclusion, this preliminary study suggests that FGS may reduce the number of surgical interventions and costs related to patients’ treatment, avoiding re‐intervention; however, further studies with larger samples are required to evaluate whether there is a statistically significant difference in the number of positive margins in patients undergoing excision with FGS or without FGS, following the current guidelines.

## CONFLICT OF INTEREST

All the authors declare that they have no conflict of interest.

## Data Availability

Data sharing not applicable to this article as no datasets were generated or analysed during the current study.

## References

[1] Nagaya T , Nakamura YA , Choyke PL , Kobayashi H . Fluorescence‐guided surgery. Front Oncol. 2017;7:314. Published 2017 Dec 22. 29312886 10.3389/fonc.2017.00314 PMC5743791

[2] Tyrrell J , Paterson C , Curnow A . Regression analysis of protoporphyrin IX measurements obtained during dermatological photodynamic therapy. Cancers (Basel). 2019;11(1):72. 10.3390/cancers11010072 PMC6356372 30634715

[3] Fritsch C , Lang K , Neuse W , Ruzicka T , Lehmann P . Photodynamic diagnosis and therapy in dermatology. Skin Pharmacol Appl Skin Physiol. 1998;11(6):358‐373. 10343206 10.1159/000029858

[4] Flores E , Yélamos O , Cordova M , et al. Peri‐operative delineation of non‐melanoma skin cancer margins in vivo with handheld reflectance confocal microscopy and video‐mosaicking. J Eur Acad Dermatol Venereol. 2019;33(6):1084‐1091. 30811707 10.1111/jdv.15491 PMC6534461

[5] Torezan LA , Festa‐Neto C . Cutaneous field cancerization: clinical, histopathological and therapeutic aspects. Ann Bras Dermatol. 2013;88:775‐786. 10.1590/abd1806-4841.20132300 PMC3798355 24173184

[6] Bobyr I , Campanati A , Consales V , et al. Ingenol mebutate in actinic keratosis: a clinical, videodermoscopic and immunohistochemical study. J Eur Acad Dermatol Venereol. 2017;31(2):260‐266. 27453064 10.1111/jdv.13831

[7] Peng X , Qian W , Hou J . 5‐aminolevulinic acid (5‐ALA) fluorescence‐guided Mohs surgery resection of penile‐scrotal extramammary Paget's disease. Biosci Trends. 2017;11(5):595‐599. 29033402 10.5582/bst.2017.01224

[8] Naidoo C , Kruger CA , Abrahamse H . Simultaneous photodiagnosis and photodynamic treatment of metastatic melanoma. Molecules. 2019;24(17):3153. 10.3390/molecules24173153 PMC6749501 31470637

[9] Walsh SB , Varma R , Raimer D , et al. Utility of Wood’s light in margin determination of melanoma in situ after excisional biopsy. Dermatol Surg. 2015;41(5):572‐578. 25915625 10.1097/DSS.0000000000000345

